# Comparison of Fetal Nuchal Fold Thickness Measurements by Two- and Three-Dimensional Ultrasonography (3DXI Multislice View)

**DOI:** 10.1155/2012/837307

**Published:** 2012-02-20

**Authors:** Leonardo da Silva Valladão de Freitas, Fernanda Silveira de Bello Barros, Rômulo Negrini, Luiz Cláudio de Silva Bussamra, Edward Araujo Júnior, Sebastião Piato, Luciano Marcondes Machado Nardozza, Antonio Fernandes Moron, Tsutomu Aoki

**Affiliations:** ^1^Department of Obstetrics and Gynecology, Medical Science College of Santa Casa of São Paulo (FCMSCSP), São Paulo, SP, Brazil; ^2^Department of Obstetrics, São Paulo Federal University (UNIFESP) - Carlos Weber Street, 956 Apartamento 113 Visage, Vila Leopoldina, 05303-000 São Paulo, SP, Brazil

## Abstract

*Purpose*. To compare the measurements of fetal nuchal fold (NF) thickness by two-dimensional (2D) and three-dimensional (3D) ultrasonography using the three-dimensional extended imaging (3DXI). *Methods*. A cross-sectional study was performed with 60 healthy pregnant women with a gestational age between 16 and 20 weeks and 6 days. The 2D-NF measurements were made as the distance from the outer skull bone to the outer skin surface in the transverse axial image in the suboccipital-bregmatic plane of the head. For the 3D we employed the 3DXI multislice view software, in which 3 × 2 tomographic planes was displayed on the screen and the distance between the tomographic slices was 0.5 mm. Maximum, minimum, mean, and standard deviation were calculated for 2D and 3D ultrasonography, as well the maximum and minimum, mean, and standard deviation for the difference between both methods. The Wilcoxon signed-rank test was used to compare the two different techniques. *Results*. 2D-NF showed a mean of thickness of 3.52 ± 0.95 mm (1.69–7.14). The mean of 3D-NF was 3.90 ± 1.02 mm (2.13–7.72). The mean difference between the methods was 0.38 mm, with a maximum difference of 3.12 mm. *Conclusion*. The NF thickness measurements obtained by 3D ultrasonography were significantly larger than those detected with 2D ultrasonography.

## 1. Introduction

The trisomy of chromosome 21 (T21) is the commonest chromosomal anomaly in newborns. The incidence is around 1 : 700 live-born infants [1, 2]. In about 95% of the cases of T21 the cause is a nondisjunction of chromosome 21 during meiosis I, which leads to the production of a triploid gamete by the maternal ovary. Robertsonian translocations and mosaicism are less common causes of that trisomy [1]. 

The phenotypic characteristics which compound T21 are highly diversified, and can affect various systems and structures. Of great clinical importance are cardiac malformations, present in 40% to 60% of the patients with T21, because they constitute in the main factors that influence the prognosis [3]. Hematologic alterations [4, 5], mental retardation [6], subnormal height, obesity [7], hearing disorders [8], ophthalmologic disorders [9], and hypothyroidism [10] can affect individuals with the trisomy.

The prenatal detection of T21 allows the allocation of the gestating woman to tertiary care centers, which have infrastructure to adequately treat eventual serious anatomical abnormalities of those newborns, particularly cardiac anomalies, improving the survival prognosis.

Ultrasound enables a better selection of gestating women eligible to a prenatal diagnosis of trisomies. Some ultrasonographic markers in the second trimester would be more frequent in T21 compromised fetuses, such as shortening of long bones (femur and humerus), intestinal hiperecogenicity, lack of nasal bone hypoplasia, and nuchal fold thickness [11–15]. Among the aforementioned markers, the nuchal fold (NF) thickness is the most sensitive and specific for T21 in the second trimester of gestation [16, 17] with sensitivity of up to 75% (average of 34%) and average rate of false positives of 0.5% (varying from 0 to 3%) [12, 13].

The studies carried out so far only evaluated the nuchal fold thickness by two-dimensional (2D) ultrasonography. Three-dimensional (3D) ultrasonography was introduced in obstetrics practice in the early 90's, and a new software called three-dimensional extended imaging (3DXI-Medison, Seoul, Korea) has been recently launched. It contains the multislice view program, which allows a simultaneous display of parallel sequences of a reference plane, in a way that the images shown on the screen are successive tomographic cuts of the 3D volume. The reference plan, the number of cuts shown on the screen, the orientation, rotation, and magnification of the image, in addition to the depth and thickness of the cut are defined according to the region of interest (ROI) [18].

The aim of this study is to compare the measurement of the NF thickness obtained by 2D and 3D ultrasonography using the multislice view program of the 3D XI software.

## 2. Materials and Methods

An observational cross-sectional study has been performed, which involved 60 normal pregnant women, with gestational ages between 16 weeks and 20 weeks and 6 days. The assessment period lasted from April 2009 to January 2010, and the patient trial has been carried out randomly. The study obtained approval from the Research Ethics Committee of the Faculty of Medical Sciences of Santa Casa de Misericórdia de São Paulo (FCMSCSP). All gestating women consented to voluntary participation and signed a consent form. 

Gestational age was determined through ultrasound performed between the 6th and 13th weeks, being assessed through the measurement of the crown-rump length (CRL). The exclusion criteria were malformations detected in previous ultrasound exam, fetal growth restriction, cystic hygroma, multiple gestation, first trimester bleeding, quantitative disturbances of the amniotic fluid (oligohydramnios and polyhydramnios), maternal clinical intercurrences, tabagism, and use of illegal drugs.

The ultrasonographic assessments were performed by a single examiner (LSVF), with 2-year experience in 3D-ultrasonography in obstetrics. The equipment used was a Sonoace X8 Live (Medison, Seoul, Korea), equipped with a multifrequencial convex volumetric transducer of automatic sweep (4–7 MHz). Only a single measurement of the NF, 2D and 3D, in each pregnant woman was performed.

**Figure 1:**
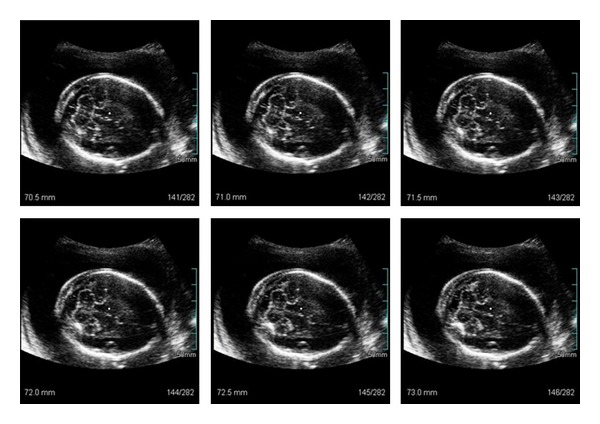
Nuchal fold thickness evaluation by three-dimensional extended imaging (3DXI) using multislice view program.

First of all, the measurement of the NF thickness was taken through 2D ultrasonography, in agreement with the currently accepted technique [11–13, 16]. After that, the 3D volume of the cephalic pole was acquired, considering as referential points the transverse diameter of the cerebellum, thalamus, and cavum of the septum pellucidum. Subsequently, the 3DXI software was activated with the multislice view program for the measurement of the NF, which was performed off line and at a different moment of the 2D measurement. The following pattern was used for the multislice view program: on the screen of the equipment 3 × 2 parallel slices of the studied plan were shown, which consist of successive tomographic slices of the 3D volume and 0.5 mm difference of depth of slice (Figure 1). Buttons 3 and 4 of the equipment were maneuvered, for the navigation between the tomographic slices, until the disappearance of the cerebellum image could be observed in one of them, on a higher plane than the one of the posterior fossa. From then on, the option 1 × 1 was selected, and the number of the image was taken. With only a single tomographic slice on the screen, button 4 of the equipment was turned clockwise or counterclockwise, depending on the fetal presentation, leading to the display of a lower plane, successively, until the sketch of the cerebellum started to show. The measurement of the NF thickness was then performed on each plane, with a difference of 0.5 mm between them, until the plane on which the habitual outline of the cerebellum was lost and the bones of the base of the skull could be observed. On average, the measurement was performed on 25 consecutive planes in each volume, considering as the nuchal fold value through the 3DXI (3D-NF) method the higher one obtained.

The data obtained was tabbed in an electronic spreadsheet of the Excel 2003 spreadsheet (Microsoft, Redmond, WA, USA) for posterior statistics analysis, which was performed with the SPSS program (Statistical Package for Social Sciences) version 17.0 for Windows. For both methods, means, standard deviations, and minimum and maximum values were calculated. For the comparative analysis between both methods used, Wilcoxon signed-rank test was performed, taking into account the magnitudes of the differences, as well as their ranks. Level of significancy (*P*) < 0.05 was used.

## 3. Results

One fetus with large NF thickness (7.0 mm at 18 weeks of pregnancy) was excluded, but the parents did not agree with the karyotype. This fetus was followed until delivery, and he did not present phenotype to T21. The age of the patients varied between 17 and 39 years old, with a mean of 25.5 years and standard deviation (SD) of 5.9 years. By stratifying the sample considering the cut-off age for patients with high risk for T21, there was a frequency of 93.3% (56) patients below of 35 years and 6.7% (4) above of 35 years. The gestational age varied between 16 weeks and 20 weeks and 4 days, with a mean of 18 weeks and 2 days and SD of 1 week and 2 days. 

The measurement of the average thickness of the NF obtained by 2D ultrasonography (2D-NF) was 3.52 mm ± 0.95 mm, varying between 1.69 and 7.14 mm, whereas the mean obtained by 3D ultrasonography (3D-NF) was 3.90 ± 1.02 mm, varying between 2.13 and 7.72 mm; those data are shown in Table 1. The mean difference among the measurements performed through both methods was 0.38 mm (*P* < 0.001), with maximum difference of 3.12 mm (*P* < 0.001) being observed. The SD of the difference among the measurements was 0.80 mm (*P* < 0.001). The variable created has the values of difference between 2D- and 3D-NF; thus that result presents itself as negative for some variables, as in some measurements the value of the 2D-NF observed was higher than the one obtained through 3DXI; those data are shown in Table 2. Due to the fact of the difference between both variables being statistically significant, we can affirm that, in general, the values of 3D-NF are effectively higher than the values of 2D-NF.

## 4. Discussion

In spite of the various applications of 3D ultrasonography in obstetrics, it has been verified through analysis of the literature that so far reports on comparative investigations between 3D and 2D ultrasonography have not been found with respect to the measurement of the NF thickness. 

In an attempt to avoid biases during the performance of the study, the maximum of homogeneity in our casuistry has been pursued, with respect to the interval between gestational ages. The chosen interval is the one currently accepted as the ideal pregnancy period for the assessment of the NF thickness, as established by Cho et al. [19].

In our study, it was observed that the measurement obtained by 3D ultrasonography was higher than the one obtained by 2D ultrasonography, datum with high statistical significancy (*P* < 0.001), being on average a difference of 0.38 mm between the aforementioned measurements. There were not clinical implications in such findings, being that the only fetus with a measurement above the 6.00 mm cut-off did not have the karyotype investigated during the prenatal period, since the parents decided not to have an amniocentesis. The NF assessment of that fetus had measurements of 7.14 mm through 2D ultrasonography and 7.72 mm through the 3DXI method. In the analysis of the neonatal results, that newborn was healthy, without any syndrome. 

Our study is the first one in the literature to evaluate the NF measurement thickness by 3D ultrasonography. Therefore, it was not possible to compare the results obtained by us with others in the literature. The study realized by Buckshee et al. [20] by 2D ultrasonography with the same methodology to measure the NF thickness of our study (axial plane) observed a mean of 3.8 mm between 18 to 20 weeks; this result is similar to these ones obtained by our study between 16 to 20 weeks, both by 2D (3.58 mm) and 3D ultrasonography (3.90 mm). These results prove the 2D ultrasonography to be still a better method to measure the NF thickness, because it is a cheap and available method to a great number of pregnant women. The 3D ultrasonography should be reserved to cases of high risk fetuses to T21 as large NF thickness (6.0 mm) and other positive marks to this trisomy. 

We understand that some factors may have an impact on the three-dimensional measurements, leading to the obtained results. 3D ultrasonography allows the plane by plane analysis of the targeted structure; thus we presume to be able to perform the highest measurement possible of the assessed structure. Furthermore, it is possible to perform the correction of the angle of the object, on three different planes, until the moment that it is totally visible, allowing the identification of the exact anatomic plane to be studied [21]. We believe that the aforementioned factors can improve the image to a more adequate measurement plane. Nevertheless, the 2D measurement is performed after the freezing of the image, being necessary, for an adequate measurement, appropriate angulation, and cut. The obtention of the ideal measurement, therefore, may be impaired by several factors, such as maternal biotype, fetal positioning, and ultrasonographic artifacts, among others.

The evidence that linear measurements through 3D ultrasonography are higher than the same measurements observed by 2D ultrasonography may have an impact on the calculation of the fetal biometry and the fetal weight estimation in fetuses that perchance have their morphology and biometric measurements performed through assessment of 3D volumes, as proposed by Benacerraf et al. [22]. In that study, the authors mention that there is discordance of at least 1 mm among 26% of the measurements of the biparietal diameters (BPD) and among 36% of the femoral lengths (FL). The difference between the two methods shown by Benacerraf et al. [22] is compatible with our results, and we believe that precaution has to be taken for the calculation of fetal weight through 3D evaluation, so that it is not overestimated, veiling cases of intrauterine growth restriction, which have important implications in obstetrics.

In developing countries such as Brazil it is very common that the pregnant women realize only second trimester scan to assess the fetal morphology and to research fetal marks to trisomies as T21. As biochemical test by *α*-fetoprotein and *β*-human chorionic gonadotropin [23, 24] is very expensive and available only in few particular centers, the prenatal ultrasound by thickness of nuchal fold is the main parameter to the screening of T21 in second trimester. In cases of high risk fetuses to T21, that is, when the NF thickness is near of superior limit (6.0 mm by 2D ultrasonography), the 3D ultrasonography technique can help the physicians to decide about an invasive procedure and counseling of parents. 

What is more difficult in using this technique in clinical practice is the long time necessary to make the measurement (mean of 180 seconds), but in cases of high risk of fetal trisomies, we believe that its use is justified.

## 5. Conclusions

In face of the results obtained with the performance of the presents study, we reached the conclusion that in the totality of the cases studied the measurements of the NF thickness obtained by 3D ultrasonography utilizing the 3DXI software were significantly higher than the ones attained by 2D ultrasonography. In cases of high risk fetuses to T21, that is, when the NF thickness is in superior limit (6.0 mm by 2D ultrasonography) associated or not with other trisomy marks, the 3D ultrasonography technique can help the physicians to decide about an invasive procedure and counseling of parents.

## Figures and Tables

**Table 1:** Comparison of nuchal fold thickness measurements by two- and three-dimensional ultrasonography methods.

Method	Mean (mm)	Standard deviation (mm)	Minimum (mm)	Maximum (mm)
2D-NF	3.52	0.95	1.69	7.14
3D-NF	3.90	1.02	2.13	7.72

NF: nuchal fold thickness; 2D: two-dimensional ultrasonography; 3D: three-dimensional ultrasonography using three-dimensional extended imaging.

**Table 2:** Difference among the nuchal fold thickness measurements by two- and three-dimensional ultrasonography.

Minimum (mm)	Maximum (mm)	Mean (mm)	Standard deviation (mm)	Significancy (*P*)
−1.09	3.12	0.38	0.80	0.001
